# BaMn^II^_2_Mn^III^(PO_4_)_3_

**DOI:** 10.1107/S1600536813023106

**Published:** 2013-08-23

**Authors:** Abderrazzak Assani, Mohamed Saadi, Ghaleb Alhakmi, Elham Houmadi, Lahcen El Ammari

**Affiliations:** a Laboratoire de Chimie du Solide Appliquée, Faculté des Sciences, Université Mohammed V-Agdal, Avenue Ibn Battouta, BP 1014, Rabat, Morocco

## Abstract

The title compound, barium trimanganese tris­(ortho­phosphate), was synthesized hydro­thermally. Its structure is isotypic with the lead and strontium analogues *A*Mn^II^_2_Mn^III^(PO_4_)_3_ (*A* = Pb, Sr). Except for two O atoms on general positions, all atoms are located on special positions. The Ba and one P atom exhibit *mm*2 symmetry, the Mn^II^ atom 2/*m* symmetry, the Mn^III^ atom and the other P atom .2. symmetry and two O atoms are located on mirror planes. The crystal structure contains two types of chains running parallel to [010]. One chain is linear and is composed of alternating Mn^III^O_6_ octa­hedra and PO_4_ tetra­hedra sharing vertices; the other chain has a zigzag arrangement and is built up from two edge-sharing Mn^II^O_6_ octa­hedra connected to PO_4_ tetra­hedra by edges and vertices. The two types of chains are linked through PO_4_ tetra­hedra into an open three-dimensional framework which contains channels parallel to [100] and [010] in which the Ba^II^ ions are located. The alkaline earth cation is surrounded by eight O atoms in the form of a slightly distorted bicapped trigonal prism.

## Related literature

For the isotypic lead and strontium analogues, see: Alhakmi *et al.* (2013*a* ▶) and (2013*b* ▶), respectively. For related structures, see: Adam *et al.* (2009 ▶); Assani *et al.* (2011*a* ▶,*b* ▶). For bond-valence analysis, see: Brown & Altermatt (1985 ▶). For the by- product phase, see: Moore & Araki (1973 ▶).

## Experimental

### 

#### Crystal data

BaMn_3_(PO_4_)_3_
*M* *_r_* = 587.07
Orthorhombic, 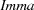

*a* = 10.3038 (7) Å
*b* = 14.0163 (11) Å
*c* = 6.7126 (4) Å
*V* = 969.44 (12) Å^3^
*Z* = 4
Mo *K*α radiation
μ = 8.39 mm^−1^
*T* = 296 K
0.29 × 0.17 × 0.13 mm

#### Data collection

Bruker X8 APEX diffractometer
Absorption correction: multi-scan (*SADABS*; Bruker, 2009 ▶) *T*_min_ = 0.164, *T*_max_ = 0.376
3968 measured reflections
811 independent reflections
732 reflections with *I* > 2σ(*I*)
*R*_int_ = 0.032

#### Refinement

*R*[*F* ^2^ > 2σ(*F* ^2^)] = 0.020
*wR*(*F* ^2^) = 0.055
*S* = 1.09
811 reflections
53 parameters
Δρ_max_ = 1.86 e Å^−3^
Δρ_min_ = −0.78 e Å^−3^

### 

Data collection: *APEX2* (Bruker, 2009 ▶); cell refinement: *SAINT* (Bruker, 2009 ▶); data reduction: *SAINT*; program(s) used to solve structure: *SHELXS97* (Sheldrick, 2008 ▶); program(s) used to refine structure: *SHELXL97* (Sheldrick, 2008 ▶); molecular graphics: *ORTEP-3 for Windows* (Farrugia, 2012 ▶) and *DIAMOND* (Brandenburg, 2006 ▶); software used to prepare material for publication: *publCIF* (Westrip, 2010 ▶).

## Supplementary Material

Crystal structure: contains datablock(s) I. DOI: 10.1107/S1600536813023106/wm2767sup1.cif

Structure factors: contains datablock(s) I. DOI: 10.1107/S1600536813023106/wm2767Isup2.hkl

Additional supplementary materials:  crystallographic information; 3D view; checkCIF report

## Figures and Tables

**Fig. 1.**
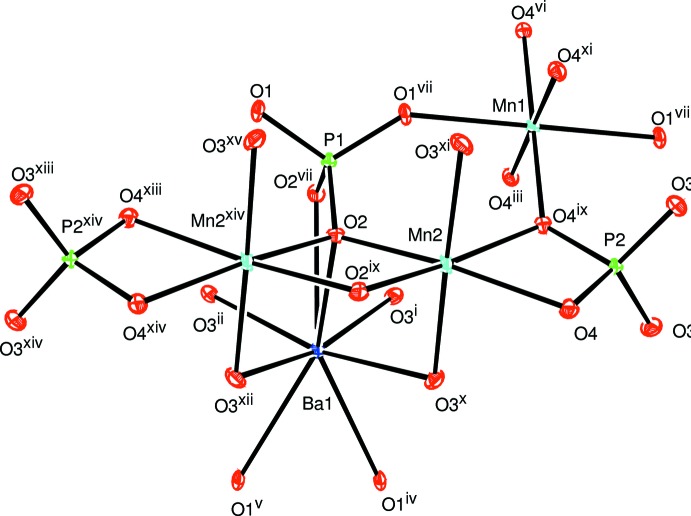
The main building units of the crystal structure of the title compound. Displacement ellipsoids are drawn at the 50% probability level. [Symmetry codes: (i) x, -y + 1, -z; (ii) -x, y - 1/2, -z; (iii) x, y - 1/2, -z; (iv) -x, -y + 1, -z; (v) x, y, z - 1; (vi) -x, -y + 1/2, z - 1; (vii) -x, -y + 1/2, z; (viii) -x + 1/2, y - 1/2, z + 1/2; (ix) x - 1/2, -y + 1/2, -z + 1/2; (x) -x + 1/2, -y + 1/2, -z + 1/2; (xi) x - 1/2, y - 1/2, z + 1/2; (xii) -x, -y, -z + 1; (xiii) -x + 1/2, -y + 1, z + 1/2; (xiv) -x + 1/2, y, -z + 1/2.]

**Fig. 2.**
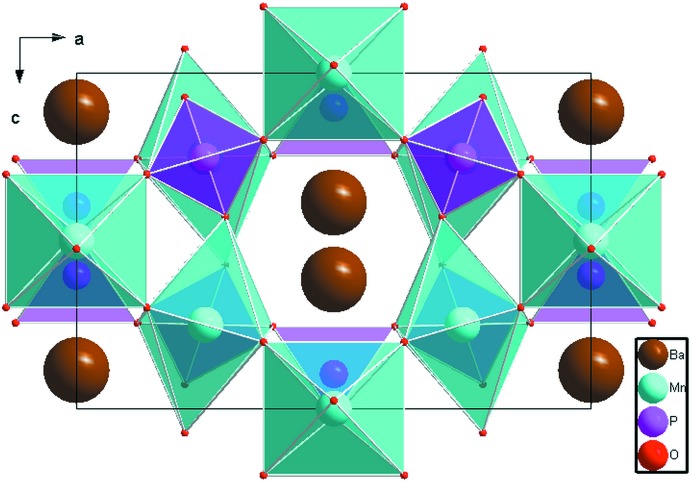
Polyhedral representation of BaMn3(PO4)3 with channels running parallel to [010].

## supplementary crystallographic information

### 1. Comment

Investigating functional compounds by means of the hydrothermal process, particularly phosphates, we have succeeded to synthesize and structurally characterize new mixed-cation orthophosphates with open frameworks, e.g. the isotypic pair Ag_2_*M*_3_(HPO_4_)(PO_4_)_2_ (*M* = Co, Ni) (Assani *et al.*, 2011**a*,b*) that is closely related to the alluaudite structure. Others investigated phosphates include compounds crystallizing in the *A*Mn^II^_2_Mn^III^(PO_4_)_3_ (*A* = Pb, Sr) structure type (Alhakmi *et al.* (2013**a*,b*) with rarely observed mixed-valent Mn^II/III^ cations (Adam *et al.*, 2009). The present article reports on synthesis and crystal structure of the isotypic barium analogue, BaMn^II^_2_Mn^III^(PO_4_)_3_.

All atoms of this structure are in special positions, except two oxygen atoms (O3, O4) in general position of space group *Imma*. The connection of the metal-oxygen polyhedra, *viz*. BaO_8_ polyhedra, MnO_6_ octahedra and PO_4_ tetrahedra is shown in Fig. 1. The framework of the crystal structure consists of two isolated PO_4_ tetrahedra linked to MnO_6_ octahedra, building two types of chains running along [010]. The first chain is formed by alternating Mn^III^O_6_ octahedra and PO_4_ tetrahedra sharing vertices. The second chain is built up from two edge-sharing Mn^II^O_6_ octahedra leading to the formation of Mn^II^_2_O_10_ dimers that are connected to two PO_4_ tetrahedra by a common edge. These two types of chains are linked together by common vertices of PO_4_ tetrahedra to form an open three-dimensional framework that delimits two types of tunnels parallel to [100] and [010] where the Ba^II^ ions are located (Fig. 2). The coordination sphere of the Ba^II^ ion is that of a bicapped trigonal prism.

Bond valence sum calculation (Brown & Altermatt, 1985) of BaMn^II^_2_Mn^III^(PO_4_)_3_ resulted in expected values (in valence units) for the ions Ba1^II^ (2.26), Mn1^III^ (3.01), Mn2^II^ (2.09), P1^V^ (4.99), and P2^V^ (4.87). The three-dimensional framework of BaMn^II^_2_Mn^III^(PO_4_)_3_ and its isotypic *A*Mn^II^_2_Mn^III^(PO_4_)_3_ (*A* = Pb, Sr) analogues, resemble that of the Ag_2_*M*_3_(HPO_4_)(PO_4_)_2_ type with *M* = Ni or Co, whereby the two Ag^+^ cations in the channels are replaced by Ba^II^, Pb^II^ or Sr^II^.

### 2. Experimental

The hydrothermal treatment of a reaction mixture of barium, manganese and phosphate precursors in a proportion corresponding to the molar ratio Ba: Mn: P = 1: 3: 3 has allowed to isolate brown block-shaped crystals corresponding to the title compound as well as a parallelepipedic colourless crystals which were identified to be the known manganese phosphate Mn_5_(HPO_4_)_2_(PO_4_)_2_^.^4H_2_O (Moore & Araki, 1973). The hydrothermal reaction was conducted in a 23 ml Teflon-lined autoclave, filled to 50% with distilled water and under autogeneous pressure at 463 K for five days.

### 3. Refinement

The highest peak and the deepest hole in the final Fourier map are at 0.82 Å and 1.00 Å away from Ba1.

### Crystal data

**Table:**

BaMn_3_(PO_4_)_3_	*F*(000) = 1088
*M**_r_* = 587.07	*D*_x_ = 4.022 Mg m^−^^3^
Orthorhombic, *I**m**m**a*	Mo *K*α radiation, λ = 0.71073 Å
Hall symbol: -I 2b 2	Cell parameters from 811 reflections
*a* = 10.3038 (7) Å	θ = 3.4–30.5°
*b* = 14.0163 (11) Å	µ = 8.39 mm^−^^1^
*c* = 6.7126 (4) Å	*T* = 296 K
*V* = 969.44 (12) Å^3^	Block, brown
*Z* = 4	0.29 × 0.17 × 0.13 mm

### Data collection

**Table:**

Bruker X8 APEX diffractometer	811 independent reflections
Radiation source: fine-focus sealed tube	732 reflections with *I* > 2σ(*I*)
Graphite monochromator	*R*_int_ = 0.032
φ and ω scans	θ_max_ = 30.5°, θ_min_ = 3.4°
Absorption correction: multi-scan (*SADABS*; Bruker, 2009)	*h* = −14→13
*T*_min_ = 0.164, *T*_max_ = 0.376	*k* = −19→20
3968 measured reflections	*l* = −9→7

### Refinement

**Table:**

Refinement on *F*^2^	0 restraints
Least-squares matrix: full	Primary atom site location: structure-invariant direct methods
*R*[*F*^2^ > 2σ(*F*^2^)] = 0.020	Secondary atom site location: difference Fourier map
*wR*(*F*^2^) = 0.055	*w* = 1/[σ^2^(*F*_o_^2^) + (0.0353*P*)^2^] where *P* = (*F*_o_^2^ + 2*F*_c_^2^)/3
*S* = 1.09	(Δ/σ)_max_ < 0.001
811 reflections	Δρ_max_ = 1.86 e Å^−^^3^
53 parameters	Δρ_min_ = −0.78 e Å^−^^3^

### Special details

**Table:**

Geometry. All s.u.'s (except the s.u. in the dihedral angle between two l.s. planes) are estimated using the full covariance matrix. The cell s.u.'s are taken into account individually in the estimation of s.u.'s in distances, angles and torsion angles; correlations between s.u.'s in cell parameters are only used when they are defined by crystal symmetry. An approximate (isotropic) treatment of cell s.u.'s is used for estimating s.u.'s involving l.s. planes.
Refinement. Refinement of *F*^2^ against all reflections. The weighted *R*-factor *wR* and goodness of fit *S* are based on *F*^2^, conventional *R*-factors *R* are based on *F*, with *F* set to zero for negative *F*^2^. The threshold expression of *F*^2^ > σ(*F*^2^) is used only for calculating *R*-factors(gt) *etc*. and is not relevant to the choice of reflections for refinement. *R*-factors based on *F*^2^ are statistically about twice as large as those based on *F*, and *R*- factors based on all data will be even larger.

### Fractional atomic coordinates and isotropic or equivalent isotropic displacement parameters (Å^2^)

**Table:**

	*x*	*y*	*z*	*U*_iso_*/*U*_eq_	
Ba1	0.0000	0.2500	−0.11499 (4)	0.01125 (10)	
Mn1	0.0000	0.5000	0.5000	0.00794 (15)	
Mn2	0.2500	0.36758 (4)	0.2500	0.01092 (13)	
P1	0.0000	0.2500	0.39677 (16)	0.0078 (2)	
P2	0.2500	0.57094 (6)	0.2500	0.00851 (17)	
O1	0.0000	0.15998 (16)	0.5237 (4)	0.0115 (5)	
O2	0.1185 (2)	0.2500	0.2553 (3)	0.0107 (4)	
O3	0.21046 (19)	0.63040 (12)	0.0721 (3)	0.0133 (3)	
O4	0.36337 (16)	0.49927 (12)	0.1983 (2)	0.0103 (3)	

### Atomic displacement parameters (Å^2^)

**Table:**

	*U*^11^	*U*^22^	*U*^33^	*U*^12^	*U*^13^	*U*^23^
Ba1	0.01480 (16)	0.01187 (15)	0.00707 (14)	0.000	0.000	0.000
Mn1	0.0102 (3)	0.0081 (3)	0.0056 (3)	0.000	0.000	−0.0001 (2)
Mn2	0.0154 (3)	0.0063 (2)	0.0110 (2)	0.000	−0.00074 (18)	0.000
P1	0.0104 (5)	0.0065 (5)	0.0066 (5)	0.000	0.000	0.000
P2	0.0121 (4)	0.0064 (4)	0.0071 (3)	0.000	0.0010 (3)	0.000
O1	0.0166 (12)	0.0049 (9)	0.0129 (11)	0.000	0.000	0.0014 (8)
O2	0.0121 (11)	0.0103 (11)	0.0098 (10)	0.000	0.0035 (8)	0.000
O3	0.0183 (8)	0.0114 (8)	0.0100 (7)	0.0027 (6)	0.0006 (7)	0.0033 (6)
O4	0.0123 (8)	0.0087 (7)	0.0100 (7)	0.0014 (6)	0.0016 (6)	0.0009 (5)

### Geometric parameters (Å, º)

**Table:**

Ba1—O1^i^	2.734 (2)	Mn2—O2	2.1337 (16)
Ba1—O1^ii^	2.734 (2)	Mn2—O2^xiii^	2.1337 (16)
Ba1—O3^iii^	2.7560 (18)	Mn2—O3^iii^	2.2006 (17)
Ba1—O3^iv^	2.7560 (18)	Mn2—O3^ix^	2.2006 (17)
Ba1—O3^v^	2.7560 (18)	Mn2—O4	2.2117 (17)
Ba1—O3^vi^	2.7560 (18)	Mn2—O4^x^	2.2117 (17)
Ba1—O2	2.769 (2)	P1—O1	1.523 (2)
Ba1—O2^vii^	2.769 (2)	P1—O1^vii^	1.523 (2)
Mn1—O4^viii^	1.9377 (17)	P1—O2^vii^	1.547 (2)
Mn1—O4^ix^	1.9377 (17)	P1—O2	1.547 (2)
Mn1—O4^x^	1.9377 (17)	P2—O3^x^	1.5119 (17)
Mn1—O4^xi^	1.9377 (17)	P2—O3	1.5119 (17)
Mn1—O1^vii^	2.248 (2)	P2—O4^x^	1.5792 (17)
Mn1—O1^xii^	2.248 (2)	P2—O4	1.5792 (17)
			
O1^i^—Ba1—O1^ii^	54.97 (9)	O4^ix^—Mn1—O1^vii^	87.51 (6)
O1^i^—Ba1—O3^iii^	111.92 (5)	O4^x^—Mn1—O1^vii^	92.49 (6)
O1^ii^—Ba1—O3^iii^	79.16 (5)	O4^xi^—Mn1—O1^vii^	87.51 (6)
O1^i^—Ba1—O3^iv^	79.16 (5)	O4^viii^—Mn1—O1^xii^	87.51 (6)
O1^ii^—Ba1—O3^iv^	111.92 (5)	O4^ix^—Mn1—O1^xii^	92.49 (6)
O3^iii^—Ba1—O3^iv^	168.02 (7)	O4^x^—Mn1—O1^xii^	87.51 (6)
O1^i^—Ba1—O3^v^	79.16 (5)	O4^xi^—Mn1—O1^xii^	92.49 (6)
O1^ii^—Ba1—O3^v^	111.92 (5)	O1^vii^—Mn1—O1^xii^	180.0
O3^iii^—Ba1—O3^v^	74.93 (8)	O2—Mn2—O2^xiii^	78.86 (10)
O3^iv^—Ba1—O3^v^	103.78 (8)	O2—Mn2—O3^iii^	84.77 (8)
O1^i^—Ba1—O3^vi^	111.92 (5)	O2^xiii^—Mn2—O3^iii^	96.38 (8)
O1^ii^—Ba1—O3^vi^	79.16 (5)	O2—Mn2—O3^ix^	96.38 (8)
O3^iii^—Ba1—O3^vi^	103.78 (8)	O2^xiii^—Mn2—O3^ix^	84.77 (8)
O3^iv^—Ba1—O3^vi^	74.93 (8)	O3^iii^—Mn2—O3^ix^	178.52 (9)
O3^v^—Ba1—O3^vi^	168.02 (7)	O2—Mn2—O4	169.28 (7)
O1^i^—Ba1—O2	142.77 (4)	O2^xiii^—Mn2—O4	107.86 (7)
O1^ii^—Ba1—O2	142.77 (4)	O3^iii^—Mn2—O4	86.16 (6)
O3^iii^—Ba1—O2	63.86 (5)	O3^ix^—Mn2—O4	92.61 (7)
O3^iv^—Ba1—O2	104.67 (5)	O2—Mn2—O4^x^	107.86 (7)
O3^v^—Ba1—O2	63.86 (5)	O2^xiii^—Mn2—O4^x^	169.28 (7)
O3^vi^—Ba1—O2	104.67 (5)	O3^iii^—Mn2—O4^x^	92.61 (7)
O1^i^—Ba1—O2^vii^	142.77 (4)	O3^ix^—Mn2—O4^x^	86.16 (6)
O1^ii^—Ba1—O2^vii^	142.77 (4)	O4—Mn2—O4^x^	66.86 (9)
O3^iii^—Ba1—O2^vii^	104.67 (5)	O1—P1—O1^vii^	111.94 (19)
O3^iv^—Ba1—O2^vii^	63.86 (5)	O1—P1—O2^vii^	110.09 (7)
O3^v^—Ba1—O2^vii^	104.67 (5)	O1^vii^—P1—O2^vii^	110.09 (7)
O3^vi^—Ba1—O2^vii^	63.86 (5)	O1—P1—O2	110.09 (7)
O2—Ba1—O2^vii^	52.33 (10)	O1^vii^—P1—O2	110.09 (7)
O4^viii^—Mn1—O4^ix^	180.0	O2^vii^—P1—O2	104.27 (19)
O4^viii^—Mn1—O4^x^	93.19 (10)	O3^x^—P2—O3	113.10 (14)
O4^ix^—Mn1—O4^x^	86.81 (10)	O3^x^—P2—O4^x^	112.12 (9)
O4^viii^—Mn1—O4^xi^	86.81 (10)	O3—P2—O4^x^	108.95 (10)
O4^ix^—Mn1—O4^xi^	93.19 (10)	O3^x^—P2—O4	108.95 (10)
O4^x^—Mn1—O4^xi^	180.0	O3—P2—O4	112.12 (9)
O4^viii^—Mn1—O1^vii^	92.49 (6)	O4^x^—P2—O4	100.99 (13)

Symmetry codes: (i) *x*, *y*, *z*−1; (ii) −*x*, −*y*+1/2, *z*−1; (iii) *x*, −*y*+1, −*z*; (iv) −*x*, *y*−1/2, −*z*; (v) *x*, *y*−1/2, −*z*; (vi) −*x*, −*y*+1, −*z*; (vii) −*x*, −*y*+1/2, *z*; (viii) *x*−1/2, *y*, −*z*+1/2; (ix) −*x*+1/2, −*y*+1, *z*+1/2; (x) −*x*+1/2, *y*, −*z*+1/2; (xi) *x*−1/2, −*y*+1, *z*+1/2; (xii) *x*, *y*+1/2, −*z*+1; (xiii) −*x*+1/2, −*y*+1/2, −*z*+1/2.
